# Alopecia areata coexisting with Hashimoto’s thyroiditis and Sjögren’s syndrome: case report of hair regrowth and selected laboratory changes during combination treatment including tofacitinib

**DOI:** 10.3389/fimmu.2026.1967270

**Published:** 2026-10-14

**Authors:** Fangjia You, Lei Wang, Ying Wang, Dingquan Yang

**Affiliations:** 1 Beijing University of Chinese Medicine, Beijing, China; 2 Department of Dermatology, National Center for Integrative Medicine, Hair Medicine Center, China-Japan Friendship Hospital, Beijing, China

**Keywords:** alopecia areata, case report, Hashimoto’s thyroiditis, Sjögren’s syndrome, tofacitinib

## Abstract

**Background:**

Alopecia areata (AA) is an autoimmune disorder characterized by transient, non-scarring hair loss and preservation of the hair follicle. Hashimoto’s thyroiditis (HT) and Sjögren’s syndrome (SS) are common systemic autoimmune disorders that may share genetic and immunological predispositions with AA. Co-occurrence of all three conditions is rarely reported.

**Case presentation:**

We report a 36-year-old female who presented with a 1-year history of progressive hair loss involving the occipital scalp margins, with a Severity of Alopecia Tool (SALT) score of 30 at treatment initiation (retrospectively revised to 15 on unblinded image review). She had a 10-year history of HT on stable levothyroxine replacement and a 6-month history of SS. Laboratory evaluation revealed elevated TSH (6.299 uIU/mL), markedly elevated TPO-Ab (922.7 IU/mL), positive anti-SSA antibodies. The patient received tofacitinib 5 mg twice daily, together with intralesional compound betamethasone, topical clobetasol propionate, and topical minoxidil. At 6 months, SALT score was 0; TPO-Ab was 255 IU/mL and Tg-Ab was 430 IU/mL; anti-SSA antibodies were negative; and TSH was 2.760 uIU/mL during continued levothyroxine replacement. Baseline and follow-up thyroid assays used different platforms (CLIA vs ECLIA), and the 6-month Tg-Ab value (430 IU/mL) was numerically higher than baseline (307.52 IU/mL); therefore, neither a biological increase nor decrease in thyroid autoantibodies can be confidently inferred from these numbers alone. Hair regrowth occurred under combination treatment and cannot be attributed to tofacitinib alone. No adverse events were reported during the available follow-up (6 months in clinic; 9 months by telephone).

**Conclusion:**

This case describes hair regrowth and selected laboratory changes observed during combination treatment including tofacitinib in a patient with AA and comorbid HT/SS. The observations are hypothesis-generating and temporal association does not establish treatment causation.

## 1 Introduction

Alopecia areata (AA) is a common non-scarring hair loss disorder with a global prevalence of approximately 2%, driven by immune privilege collapse at the hair follicle and subsequent CD8^+^ T lymphocyte-mediated autoimmune attack (1, 2). Genetic studies have identified shared susceptibility loci across autoimmune diseases, including CTLA-4, PTPN22, and the IL-2/IL-21 pathway, which converge on JAK-STAT signaling as a common mechanistic node (3–5). CTLA-4 and PTPN22 encode key regulators of T-cell activation threshold, and their polymorphisms have been associated with AA risk in multiple populations (6, 7). In AA, this pathway mediates follicular attack, while also promoting the Th17/Treg imbalance observed in lesional scalp (3, 8).

Hashimoto’s thyroiditis (HT) and Sjögren’s syndrome (SS) are two prevalent systemic autoimmune diseases. HT is characterized by thyroid-specific autoantibody production and lymphocytic infiltration of the thyroid gland, leading to hypothyroidism (9); SS predominantly involves exocrine glands, manifesting as xerostomia and xerophthalmia with multisystem autoantibody positivity (10). Epidemiological and genetic studies have demonstrated significant comorbidity between AA and both HT and SS, with shared susceptibility loci including CTLA-4, PTPN22, and IL-2/IL-21 pathway genes, as well as converging Th1/Th17-related inflammatory cascades (3, 11–13).

Conventional management strategies for AA include topical and intralesional corticosteroids, contact immunotherapy, and systemic corticosteroids or immunosuppressants. In patients with coexisting systemic autoimmune diseases, these approaches may not simultaneously address cutaneous and systemic manifestations. Janus kinase (JAK) inhibitors such as baricitinib have demonstrated efficacy in AA by blocking multiple pathogenic cytokine signals (14). Their systemic immunomodulatory potential in patients with coexisting autoimmune conditions warrants further investigation. Herein, we describe a case of AA coexisting with HT and SS in which hair regrowth and selected laboratory changes were observed during combination treatment including tofacitinib.

## 2 Case report

### 2.1 Clinical timeline

#### 2.1.1 Alopecia areata

A 36-year-old female presented to our dermatology clinic in December 2025 with a 1-year history of progressive hair loss localized to the occipital region. The initial lesion began as a coin-sized alopecic patch, which gradually expanded along the occipital scalp margins, forming a band-like pattern; the temporal and prefrontal hairline was completely preserved. The SALT score was initially recorded as 30 at the time of clinical presentation. During manuscript preparation, the original clinical and trichoscopic images were retrospectively re-evaluated by the treating dermatologist against the SALT regional weighting system (top 40%, posterior 24%, each side 19%). This re-evaluation, which was not blinded, revised the baseline score to 15 and the 3-month score to 10. The discrepancy between the original and revised scores is acknowledged. The 6-month score was 0. Because the originally documented score of 30 informed the decision to initiate systemic therapy, both the contemporaneous and retrospectively revised values are reported here. The alopecic patches demonstrated well-demarcated borders, smooth skin surfaces without scaling or erythema. Trichoscopic examination at baseline showed a smooth scalp surface with the presence of follicular openings and yellow dots, and no signs of inflammation; no black dots, broken hairs, or exclamation-mark hairs were documented. Eyebrows, eyelashes, and body hair were spared. She denied recent psychological trauma or significant stress events. Nail examination revealed no abnormalities, including no pitting, trachyonychia, onycholysis, or other onychodystrophic changes. The patient had received Chinese herbal decoction combined with acupuncture and cupping for two months, without satisfactory improvement. Progressive enlargement of the alopecic area caused a degree of psychosocial burden.

#### 2.1.2 Hashimoto’s thyroiditis

The patient reported a 10-year history of HT diagnosed at an outside hospital, with laboratory findings of markedly elevated TPO-Ab and Tg-Ab. She had been maintained on levothyroxine 25 μg daily; however, TSH levels remained above the upper reference limit despite replacement, consistent with subclinical hypothyroidism.

#### 2.1.3 Sjögren’s syndrome

Six months prior to presentation, the patient developed progressive xerostomia and xerophthalmia. Laboratory evaluation at a local hospital revealed positive anti-SSA antibodies. Labial gland biopsy demonstrated focal lymphocytic sialadenitis with a focus score ≥1/4 mm². According to the 2016 ACR/EULAR classification criteria for SS (15), the patient scored 3 points for positive anti-SSA antibodies and 3 points for focal lymphocytic sialadenitis (focus score ≥1/4 mm²), achieving a total score of 6 (≥4), thereby establishing a definite diagnosis of SS. She had not received immunosuppressive or biologic therapy and managed sicca symptoms with sodium hyaluronate eye drops.

#### 2.1.4 Current presentation and diagnosis

At presentation to our clinic, physical examination revealed a continuous alopecic band measuring 3–5 cm in width along the occipital hairline. The affected scalp was smooth with presence of follicular openings; pull test was negative. No erythema, scaling, or scarring was observed. Systemic skin and nail examinations were unremarkable.

Baseline laboratory findings (December 2025):

Thyroid function: TSH 6.299 uIU/mL, FT4 15.49 pmol/L (subclinical hypothyroidism, consistent with inadequate levothyroxine replacement);
Thyroid autoantibodies: TPO-Ab 922.7 IU/mL, Tg-Ab 307.52 IU/mL (markedly elevated);
SS-related antibodies: anti-SSA (+), anti-SSA52 (++), anti-SSB (−), anti-dsDNA (−);
Inflammatory markers: ESR 5 mm/h;
Other tests: Complete blood count, renal and hepatic function, lipid profile shown in **Table 1**; coagulation profile, immunoglobulin profile and complement C3/C4 were within normal limits. Anti-TB antibody test was negative.
Thyroid ultrasound: Diffuse thyroid disease with a right-lobe cystic-solid nodule (ACR-TIRADS 3).
Final diagnosis: Based on the hair loss, documented HT and SS history, and laboratory findings, the patient was diagnosed with (1): AA (SALT 30 at treatment initiation; retrospectively revised to 15) (2); HT; and (3) SS.

**Table 1:** Longitudinal changes in laboratory parameters before and after combination treatment including tofacitinib.

Parameter	Baseline (2025.12)	3 Months (2026.03)	6 Months (2026.06)	Platform and reference range	
WBC (10^9/L)	6.75	5.66	6.94	3.5-9.5	
NEU (10^9/L)	4.18	3.29	4.13	1.8-6.3	
RBC (10^12/L)	4.66	4.54	4.62	3.8-5.1	
PLT (10^9/L)	445	500	469	125-350	
HGB (g/L)	146	148	147	115-150	
ALT(U/L)	68.7	31	24	7-40	
AST(U/L)	41.6	19	18	13-35	
Cr (umol/L)	52	65	60	41-73	
TG (mmol/L)	1.39	Not available	Not available	0-1.7	
TC (mmol/L)	5.08	Not available	Not available	0-5.2	
TSH(uIU/mL)	6.299	7.590	2.760	CLIA:0.35-5.1; ECLIA :0.270-4.200	
FT4(pmol/L)	15.49	14.2	14.6	CLIA:11.2-23.81; ECLIA :12.00-22.00	
TPO-Ab(IU/mL)	922.7	303	255.00	CLIA:0-9; ECLIA :<34.00	
Tg-Ab(IU/mL)	307.52	642	430	CLIA: 0–4; ECLIA: <115.00	
Anti-SSA	+	+	–	Semiquantitative; cutoff not available	
Anti-SSA52	++	+	Not available	Semiquantitative; cutoff not available
Anti-TB	–	Not available	Not available	Semiquantitative; cutoff not available

TSH, FT4, TPO-Ab, Tg-Ab used chemiluminescence immunoassay (CLIA) at baseline and electrochemiluminescence immunoassay (ECLIA) at follow-up; anti-SSA/anti-SSA52 used line immunoassay (LIA) throughout. Anti-SSA may refer to Ro60, or unclassified mixed reactivity.

### 2.2 Therapertic intervention and follow-up

At baseline (December 2025):

Pretreatment assessment documented a negative anti-TB antibody test (Semi-quantitative colloidal gold; December 15, 2025), and no history of hepatitis infection. The patient reported no recent vaccination and no current plans for pregnancy. Baseline platelet count was elevated and ALT/AST were mildly elevated. These pre-existing abnormalities were noted but did not influence the treatment decision. The patient had a 1-year progressive course, failured previous AA treatment, and psychosocial impairment. After comprehensive discussion of risks and benefits, the patient was initiated on oral tofacitinib 5 mg twice daily in December 2025. At the time of treatment initiation, the clinically recorded SALT score was 30; this was later revised to 15 on retrospective unblinded review. The decision to initiate systemic therapy was based on the originally recorded severity, the progressive course, failure of prior Chinese medicine and acupuncture treatment, psychosocial burden, and coexisting systemic autoimmunity.

Alternatives considered included intralesional corticosteroid monotherapy and topical immunotherapy. Rheumatology and endocrinology consultations were not documented, and the treatment decision was made by the dermatology team. Tofacitinib is not approved by the U.S. Food and Drug Administration for alopecia areata, the patient was informed of the off-label nature of the treatment, and provided consent. Levothyroxine dose was maintained at 25 μg daily throughout the treatment period. Concomitant local therapy comprised two sessions of intralesional compound betamethasone at baseline and at 3 months. The intralesional corticosteroid injection was performed as follows: 1 mL of compound betamethasone injection, containing betamethasone dipropionate 5 mg and betamethasone sodium phosphate 2 mg, was diluted 1:1 with lidocaine hydrochloride injection and administered via multi-point intralesional injection. The injection points were spaced approximately 1 cm apart, and the injection depth was 0.3–0.4 cm, targeting the deep dermis to the superficial subcutaneous fat layer. Approximately 0.1 mL was injected per point. The syringe was shaken every 2–3 injections to maintain uniform suspension of the mixture. 2% minoxidil liniment, and compound clobetasol propionate ointment applied nightly until 6 months. The 3-month laboratory measurements were obtained before the second betamethasone injection.

At 3 months (March 2026):

Cutaneous response: visible vellus hair regrowth was observed in the alopecic areas, most notably at the occipital margins (**Figure 1**); SALT score was 10.

**Figure 1:**
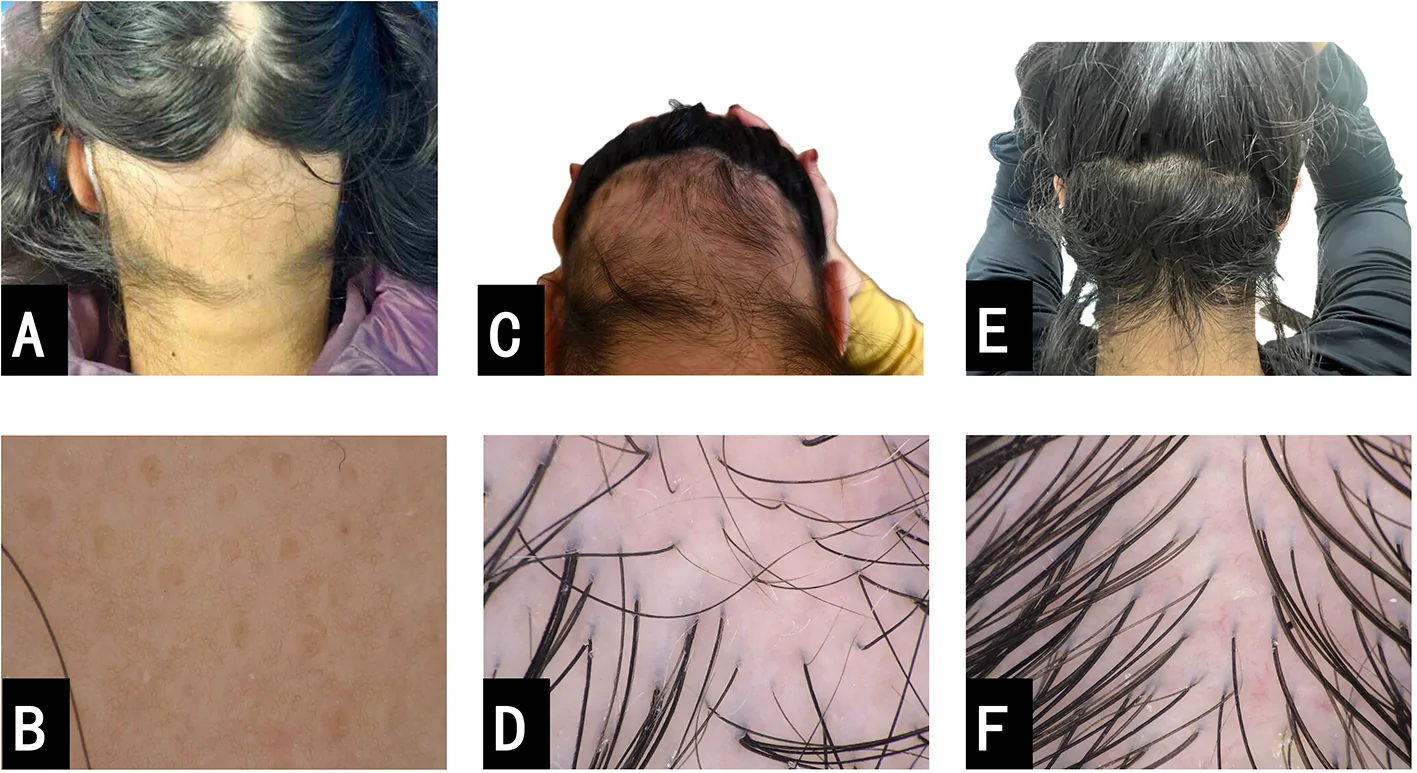
Clinical and trichoscopic (×40) findings of alopecia areata before and after combination treatment including tofacitinib. **(A, B)** Baseline (December 2025): Clinical photograph **(A)** showing a band-like alopecic area restricted to the occipital scalp margins, with preservation of the temporal hairline. Trichoscopic image **(B)** showing a smooth scalp surface with visible follicular openings, yellow dots, and no signs of inflammation. SALT score: 30 at treatment initiation (retrospectively revised to 15). **(C, D)** At 3 months (March 2026): Clinical photograph **(C)** showing visible vellus hair regrowth within the previously alopecic area. Trichoscopic image **(D)** showing newly formed vellus hairs emerging from the follicular openings. SALT score: 25 at the time of assessment (retrospectively revised to 10). **(E, F)** At 6 months (June 2026): Clinical photograph **(E)** showing terminal hair regrowth covering the previously affected area. Trichoscopic image **(F)** showing normal-density terminal hairs with visible follicular openings. SALT score: 0.

Laboratory findings: TSH 7.590 uIU/mL, FT4 14.2 pmol/L; TPO-Ab 303 IU/mL, Tg-Ab 642 IU/mL; anti-SSA (+) and anti-SSA52 (+). (**Table 1**)

At 6 months (June 2026):

Cutaneous response: terminal hair regrowth covering the previously affected area; SALT score was 0 (**Figure 1**). The patient reported high satisfaction with no new alopecic patches.

Laboratory findings: TSH 2.760 uIU/mL, FT4 14.6 pmol/L; TPO-Ab 255 IU/mL, Tg-Ab 430 IU/mL; anti-SSA antibodies negative (**Table 1**).

Symptomatic improvement: Subjective reduction in xerostomia and xerophthalmia was noted without additional symptomatic therapy.

At 9 months (September 2026):

At a telephone follow-up conducted, the patient reported continued hair growth and no recurrence of alopecic patches. The current tofacitinib dose was reduced to 5mg once daily. No new adverse events were reported. This follow-up was conducted by telephone; clinical examination, trichoscopy, and laboratory testing were not performed at this time point.

### 2.3 Safety assessments

Complete blood count, renal function, and hepatic function were assessed at baseline and at 3 and 6 months (**Table 1**). Platelet counts were above the reference range at all three time points. Because platelet counts were elevated at baseline before tofacitinib initiation, this abnormality was pre-existing rather than treatment-emergent. ALT and AST were elevated at baseline, and normalized at 3 months and 6 months (**Table 1**). Lipid profile was available at baseline only (**Table 1**), values at 3 and 6 months were not available. No adverse events were reported by the patient during the available follow-up.

### 2.4 Integrated treatment-assessment timeline

At baseline (December 2025), SALT was 30 (retrospectively revised to 15), thyroid laboratory tests were obtained on CLIA, and SS-related laboratory tests used LIA. Tofacitinib 5 mg twice daily was initiated together with intralesional compound betamethasone (1 mL, containing betamethasone dipropionate 5 mg and betamethasone sodium phosphate 2 mg, multi-point), topical 2% minoxidil, and topical clobetasol propionate.

At 3 months (March 2026), before the second betamethasone injection, SALT was 25 (retrospectively revised to 10) and thyroid parameters were re-measured on ECLIA, SS-related laboratory tests used LIA. Oral and topical treatments were continued as before.

At 6 months (June 2026), SALT was 0, anti-SSA was negative used LIA, and thyroid parameters were re-measured on ECLIA. Tofacitinib 5 mg twice daily and levothyroxine 25 μg daily were continued. Topical treatments were discontinued after 6 months.

At 9 months (September 2026), a telephone follow-up was conducted. The patient reported continued hair growth and no recurrence of alopecic patches. Tofacitinib was reduced to 5 mg once daily in September 2026 by the treating dermatologist, based solely on the patient-reported sustained response during this telephone follow-up. No objective clinical assessment, trichoscopy, SALT scoring, or laboratory monitoring was performed at the time of dose reduction.

## 3 Discussion

We report a case of AA coexisting with HT and SS in which hair regrowth and selected laboratory changes were observed during combination treatment including tofacitinib. The temporal relationship—hair regrowth within 3 months of treatment initiation after a 1-year progressive course, together with changes in systemic autoantibody measurements—is described here as a temporal association. Spontaneous fluctuation of AA, concomitant local therapy, and assay platform changes cannot be excluded as alternative explanations. These observations are hypothesis-generating and do not establish that tofacitinib modulated a shared immunopathological axis in this patient.

AA is a T cell–mediated autoimmune disease targeting the hair follicle. IFN-γ signals through JAK1/JAK2, while IL-2 and IL-15 signal via JAK1/JAK3 (16); this cascade drives MHC class I upregulation on follicular epithelium and activates NKG2D^+^CD8^+^ T cells (17). In HT, Th1 cells predominate and IFN-γ induces aberrant MHC class II expression on thyrocytes, with Tfh cells promoting B-cell maturation and TPO-Ab/Tg-Ab production (18). In SS, IFN-γ drives salivary gland epithelial injury through JAK/STAT1 (19). Previous meta-analysis have confirmed significant comorbidity between AA and both HT and SS. The pooled risk of HT in AA patients was increased approximately 2.15-fold in one meta-analysis (11) and 4.31-fold in another (20), while SS risk was elevated approximately 1.6-fold (20). However, these studies predominantly lacked dynamic treatment-response tracking.

Multiple autoimmune syndrome (MAS) represents a clinical entity in which three or more autoimmune diseases coexist in the same individual (21). MAS type 3 classically includes thyroiditis, vitiligo, and alopecia areata (21, 22). Our patient fulfills the general concept of MAS, though not the classic type 3 triad, presenting with HT, SS, and AA. Epidemiological studies have reported increased comorbidity between AA and both HT and SS, with shared susceptibility loci including CTLA-4, PTPN22, and IL-2/IL-21 pathway genes. These observations indicate that autoimmune diseases can cluster within individuals. In this patient, HT, SS, and AA developed sequentially over a decade, a pattern consistent with autoimmune clustering. This clustering has important clinical implications: patients with one autoimmune disease, particularly those with organ-specific autoantibodies, warrant vigilant screening for additional autoimmune conditions. In patients with AA, clinical assessment for symptoms suggestive of thyroid disease may be warranted, with targeted testing such as TSH and, where clinically indicated, thyroid autoantibodies or anti-SSA/SSB. Conversely, patients with established HT or SS who develop hair loss should be evaluated for AA. The extent of screening should be guided by clinical presentation rather than applied universally.

Anti-SSA antibodies can precede clinical autoimmune disease by years, with a median onset of 4.3–5.1 years before Sjögren’s syndrome diagnosis (23). In our patient, anti-SSA was negative at 6 months on a single semiquantitative assay; this result was not confirmed by repeat testing. A single negative result does not establish SS remission or eliminate future autoimmune risk. No mechanism underlying this change can be inferred from the available data. Recent clinical evidence supports the potential of tofacitinib in Sjögren’s disease. Liu et al. (24) reported a retrospective cohort and a prospective cohort of patients with Sjögren’s disease treated with tofacitinib. The retrospective cohort included 112 patients and significant reductions in ESSDAI were observed. The prospective cohort of 10 patients with SjD demonstrated significant reductions in ESSDAI and ESSPRI scores at 6 months with tofacitinib 5 mg twice daily, along with reduced Tfh and Tph cell frequencies. However, ESSDAI and ESSPRI were not assessed in our patient, limiting direct comparison.

The extent of scalp involvement in AA carries prognostic significance (25). The SALT score documented at treatment initiation was 30 (retrospectively revised to 15), indicating moderate involvement by the contemporaneous assessment. No inference regarding prognosis can be drawn from this single observation. Furthermore, nail involvement is a common yet often overlooked manifestation of AA, and its presence has been associated with more severe disease (26). The response of nail involvement to JAK inhibition is an emerging area of interest, with preliminary reports suggesting potential benefit (27). In our patient, nail examination revealed no abnormalities at baseline, and no nail changes developed during the 6-month follow-up period.

Topical and intralesional therapies administered in this patient included intralesional compound betamethasone, 2% minoxidil liniment, and compound clobetasol propionate ointment. Adverse effects associated with intralesional corticosteroids include transient pain during injection, skin atrophy, and telangiectasia with repeated treatments. Topical clobetasol propionate may cause skin atrophy, folliculitis, and telangiectasia. Topical minoxidil is generally well tolerated with minimal local irritation. In this patient, local adverse effects were not observed during the available follow-up period.

At 6 months, SALT was 0, TSH was 2.760 uIU/mL during continued levothyroxine replacement, TPO-Ab was 255 IU/mL, Tg-Ab was 430 IU/mL, and anti-SSA was negative. These laboratory values of thyroid function were obtained on a different platform from baseline (ECLIA vs CLIA) with non-identical reference ranges; direct numerical comparison across time points is therefore limited. In particular, the 6-month Tg-Ab value (430 IU/mL) was numerically higher than baseline (307.52 IU/mL), and neither a biological increase nor decrease can be confidently inferred. TSH normalization during continued levothyroxine replacement does not establish HT resolution, and TPO-Ab and Tg-Ab remained positive. A single negative anti-SSA result does not establish SS remission or eliminate future autoimmune risk. These findings are described as temporal associations and are consistent with recent reports of JAK inhibitor immunomodulation in systemic lupus erythematosus (28–30) and dermatomyositis (31), but they do not establish a shared mechanism in this patient.

In this patient, ALT and AST were elevated at baseline, and normalized at 3months and 6 months. The baseline elevation preceded tofacitinib initiation and could not be definitively attributed to tofacitinib given the pre-existing abnormality. Platelet counts were above the reference range at all three time points. Because platelet counts were elevated at baseline before tofacitinib initiation, a direct drug effect cannot be inferred from these data. Platelet counts should be monitored in patients receiving JAK inhibitors, and the clinical significance of persistent mild thrombocytosis in this patient remains uncertain.

This report has several limitations. First, the patient received concomitant intralesional betamethasone, topical clobetasol propionate, and topical minoxidil in addition to tofacitinib; hair regrowth cannot be attributed to tofacitinib alone. Second, spontaneous fluctuation of AA cannot be excluded, particularly given the relapsing–remitting nature of the disease. Third, SALT assessment was performed by the treating dermatologist without blinded central review. Fourth, baseline and follow-up thyroid assays used different platforms (CLIA vs ECLIA), limiting direct numerical comparison. Fifth, several measurements were not obtained, including lipid profile, 6-month anti-Ro52, and objective glandular testing. Sixth, follow-up was limited to 6 months, with the latest assessment conducted by telephone. Seventh, baseline thrombocytosis and transaminase elevations were not further evaluated with repeat testing, hepatitis serology, medication/alcohol history, imaging, or specialist consultation. These limitations preclude causal inference and limit the generalizability of the observations.

This case is descriptive and hypothesis-generating. No clinically actionable management strategy can be derived from a single case. Clinicians evaluating patients with AA and coexisting systemic autoimmune disease may consider clinical assessment for symptoms suggestive of thyroid or sicca disease, with targeted testing guided by clinical presentation.

## 4 Conclusion

This case describes hair regrowth and selected laboratory changes observed during combination treatment including tofacitinib in a patient with AA and comorbid HT/SS. Hair regrowth occurred under combined systemic and local therapy and cannot be attributed to tofacitinib alone. Spontaneous fluctuation of AA, systemic absorption of intralesional corticosteroids, and assay platform changes cannot be excluded. The observations are hypothesis-generating; no clinically actionable management strategy can be derived from this single case. Controlled studies are needed.

## Data Availability

The original contributions presented in the study are included in the article/supplementary material. Further inquiries can be directed to the corresponding author.
